# Study on the Microstructure, Mechanical Properties, and Corrosion Behavior of 900 °C-Annealed CoCrFeMnNiSix (X = 0, 0.3, 0.6, 0.9) High-Entropy Alloys

**DOI:** 10.3390/e26110897

**Published:** 2024-10-23

**Authors:** Chunxia Jiang, Rongbin Li, Zaikang Zong, Wenge Li, Yong Zhang, Tongyao Li

**Affiliations:** 1 School of Materials Science and Engineering, Shanghai Dianji University, Shanghai 201306, China; jiangcx@sdju.edu.cn (C.J.); 18856622186@139.com (Z.Z.); 2 Merchant Marine College, Shanghai Maritime University, Shanghai 201306, China; liwenge66@163.com; 3 State Key Laboratory for Advanced Metals and Materials, University of Science and Technology Beijing, Beijing 100083, China; drzhangy@ustb.edu.cn; 4 Management College, Wuhan Donghu University, Wuhan 430212, China; y15896039166@126.com

**Keywords:** CoCrFeMnNiSix HEA, silicon addition, heat treatment, microstructure, mechanical properties, corrosion

## Abstract

In this study, a series of CoCrFeMnNiSix (x = 0, 0.3, 0.6, 0.9) high-entropy alloys (HEAs) were prepared by suspension melting of cold crucible, annealed at 1000 °C, and then quenched at 900 °C. The changes in the microstructure of the HEAs after the addition of Si were analyzed using X-ray diffraction (XRD), metallographic microscope, scanning electron microscopy (SEM) with energy dispersive spectroscopy (EDS), and electron backscatter diffraction (EBSD). The hardness, room-temperature friction, and wear behavior, room-temperature compressive properties, and corrosion resistance of the annealed CoCrFeMnNiSix HEAs were also studied. The results show that when the Si content is 0 and 0.3, the annealed CoCrFeMnNiSix HEA exhibits a single face-centered cubic (FCC) structure. As the silicon content increases, a face-centered orthorhombic (FCO) phase appears. At a Si content of 0.9, a hexagonal close-packed (HCP) phase is observed. After heat treatment, the hardness of the CoCrFeMnNiSix HEAs increases continuously with the addition of Si. The HEA with a Si content of 0.9 achieves the highest hardness of 974.8 ± 30.2 HV. The HEA with a Si content of 0.6 reaches the highest compressive strength and yield strength, which are 1990.3 MPa and 1327.5 MPa. When the Si content is 0.9, the HEA shows the smoothest surface after wear, with the best wear resistance, achieving a value of 0.21 mm^−1^. In the CoCrFeMnNiSix HEAs after 900 °C heat treatment, the HEA with a Si content of 0.6 exhibits the lowest self-corrosion current density of 0.23 µA/cm^2^ and the highest pitting potential of 157.65 mV, indicating the best corrosion resistance.

## 1. Introduction

Traditional alloy design theory suggested that multi-principal element alloys would form multiple intermetallic compounds. In 1995, Yeh et al. proposed the concept of multi-principal element alloys, suggesting that they tend to form solid solution structures rather than complex intermetallic compounds. In 2004, Professor Yeh and Professor Cantor formally introduced the concept of HEAs. They utilize the characteristics of high mixing entropy by combining five or more elements in an equimolar (or near-equimolar) ratio, where the content of each principal element is within the range of 5 at.% ≤ n ≤ 35 at.% [1,2,3]. HEAs are distinguished by their high mixing entropy and the inclusion of multiple principal elements, which result in unique characteristics such as the high-entropy effect, lattice distortion, sluggish diffusion, and the “cocktail” effect [4,5,6]. These alloys exhibit remarkable properties, including superior strength, wear resistance, corrosion resistance, and stability under extreme conditions. Their potential applications span diverse fields, including aerospace, semiconductors, transportation, marine exploration, superconductivity, and energy storage. Current research on HEAs primarily focuses on adjusting the proportion of metal elements in the alloy to influence its properties. Recently, scholars have discovered that adding some non-metallic elements with relatively small atomic sizes can enhance the performance of the HEAs [7,8,9,10]. This discovery not only further expanded the compositional design space of HEAs but also reduced the cost of HEA preparation. Some non-metallic atoms with smaller atomic radii primarily exist in the solid solution as interstitial atoms. This mismatch in atomic radii and elastic moduli between the principal elements and the added elements results in significant lattice distortion, which unexpectedly enhances the strength of HEAs [11,12]. Additionally, the addition of non-metallic interstitial atoms contributes to solid solution strengthening, grain refinement, and second phase strengthening, all of which can impact the microstructure and properties of the HEAs [13,14]. For instance, the addition of B to the AlCoCrFeNi HEAs [15] significantly improves its soft magnetic properties and hardness. Similarly, incorporating an appropriate amount of C into the CoCrFeMnNi HEA [16] enhances its strength, though it slightly reduces ductility. The addition of C to the CoCrFeMnNi HEAs [17] improves both its strength and ductility at room and low temperatures. The corrosion resistance of the AlCoCrFeNi HEA is significantly improved by adding varying amounts of C [13]. Specifically, when the carbon content reaches 0.4, the passive current density decreases to 2.57 μA/cm^2^, which is 1.6 times lower than that of the carbon-free HEA. Yanchong Xie [10] used mechanical alloying followed by vacuum hot pressing sintering to prepare a CoCrFeNiMn HEA, investigating the effect of N addition on its mechanical properties. The results indicated that the addition of 0.1 at.% N increased the Vickers hardness of the HEA to 468 HV. Additionally, the yield strength and ultimate compressive strength improved by 203 MPa and 115 MPa, respectively. This enhancement is attributed to the strengthening effect of nitrogen, which acts to refine grains and enhance solid-solution hardening, leading to an overall improvement in the mechanical performance of the HEA. Studies have shown that adding a certain amount of Si to HEAs can effectively improve their mechanical properties, corrosion resistance, and electromagnetic properties [18,19,20,21]. Research indicates that the addition of C and Si to FeMnCoCr HEAs [22], the non-metallic atoms are completely dissolved in the matrix, and the addition of C and Si significantly increases the strength of HEA. The addition of Si to the FeCoNiAlSix HEA transforms its crystal structure from FCC and body-centered cubic (BCC) structure to a single BCC phase [18]. As the Si content increases, the thermal expansion behavior of the FeCoNiAlSix HEA is reduced, particularly at high temperatures. This indicates a synergistic effect in which Si plays a crucial role in enhancing the stability of the HEA in elevated temperature environments, making it more suitable for applications requiring thermal stability.

Many research results currently indicate that appropriate heat treatment has a significant impact on regulating the microstructure and properties of HEAs. Adopting an appropriate heat treatment process can optimize the proportion of various strengthening mechanisms in the HEA, eliminate harmful phases, and enhance the overall performance of the HEA. However, previous research has primarily focused on studying the effects of different heat treatment temperatures on the performance changes of HEAs [23,24,25,26], with more attention given to the performance changes of cast alloys. There is still relatively little research on the characteristics of HEAs after homogenization annealing followed by quenching treatment. In this study, CoCrFeMnNiSix (x = 0, 0.3, 0.6, 0.9) HEAs were prepared using the cold crucible levitation melting method, followed by annealing at 1000 °C and quenching at 900 °C. The changes in the microstructure of the HEAs after the addition of silicon were analyzed using XRD, SEM with EDS, and EBSD. The hardness, room-temperature friction and wear behavior, room-temperature compressive properties, and corrosion resistance of the annealed CoCrFeMnNiSix HEAs were also studied.

## 2. Materials and Methods

**Figure 1:**
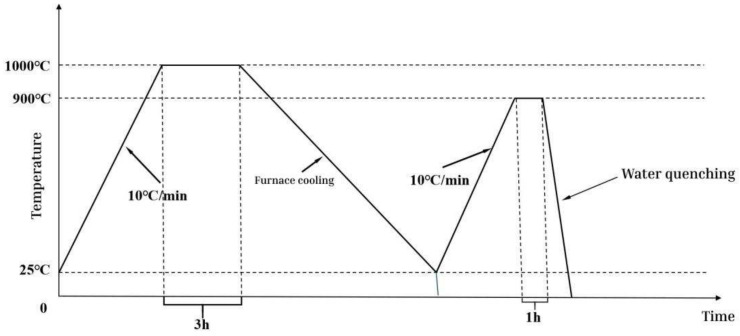
Heat treatment flowchart.

In this experiment, the CoCrFeMnNiSix HEAs were prepared using the levitation melting method under an argon atmosphere. Each sample was remelted at least five times. The as-cast HEAs with different compositions were subjected to heat treatment. The sealed samples were placed in an N61/H-type muffle furnace, where the temperature was raised to 1000 °C and held for 3 h for homogenization annealing to eliminate compositional segregation formed during casting. Homogenization annealing is conducted at 1000 °C near the phase transformation point of the alloy. Quenching treatment at 900 °C can achieve phase stability and enhance the properties of the alloy [27,28,29]. The furnace was then cooled to room temperature. Subsequently, the temperature was raised to 900 °C and held for 1 h. To preserve the microstructure at 900 °C, the samples were immediately quenched in water. The heat treatment process is shown in Figure 1.

A BRUKER D8 XRD (BRUKER, Billerica, MA, USA) with a Cu target was used for phase analysis. The diffraction angle (2θ) was scanned in the range of 20° to 100° at a scanning speed of 3°/min. The surface oxide layer has been removed before XRD testing. The samples were ground using SiC sandpaper, then polished with diamond polishing slurry. After corrosion using a mixed solution of nitric acid and hydrochloric acid in a volume ratio of 1:3, The microstructure of the HEA was examined using a LEICA DM4000 M LED metallographic microscope, and a HITACHI S-3400N tungsten filament SEM equipped with an EDS to determine elemental composition. The mechanically polished samples were immersed in a solution of 10% perchloric acid and 90% ethanol for electrolytic polishing at 10 V for 15 s, followed by EBSD testing. The EBSD data were exported to TSL-OIM™ (7.3.1.1) software for analysis. The step of EBSD testing is 35 μm.

Hardness testing was performed using an HXD-1000 Digital Micro Vickers Hardness Tester (Shanghai Optical Instrument Factory, Shanghai, China), applying a test load of 0.98 N with a dwell time of 15 s. At least 10 points were tested on each sample, with a spacing of 50 μm between test points. The room-temperature compressive properties of the HEA were tested using a Gleeble 3180 Thermal Simulation Testing Machine (from Dynamic Systems Inc., New York, NY, USA). The sample size was a Φ3 mm × 5 mm cylinder, with a compression strain rate of 1 × 10^−3^ s^−1^ and a total strain of 0.6. The test was repeated three times. The room-temperature friction and wear tests of the HEA were conducted on an RTEC friction and wear tester. A Si₃N₄ ceramic ball with a diameter of 8 mm was used as the counterpart for the friction test, while the sample was prepared as a disc with dimensions of Φ30 mm × 3 mm. The friction radius was set at 4 mm, and the test was carried out at a rotational speed of 120 rpm under a load of 30 N for 30 min.

Electrochemical corrosion testing was performed on heat-treated HEAs samples with different compositions. The experiments were conducted using a VMP-3E 16-channel Electrochemical Workstation (Bio-Logic, Cessy near Paris, France), connected to a three-electrode system. The reference electrode (SCE) was a saturated calomel electrode, and the prepared alloy samples were used as the working electrode, with a pure Pt sheet as the counter electrode. The specific experimental procedures and parameters were as follows: (1) the working electrode was anodically reduced to a potential of −0.8 V for 5 min to remove the oxide layer formed in air. (2) An open-circuit potential (OCV) test was conducted by placing the sample in a 3.5 wt.% sodium chloride solution and waiting for 30 min until the open-circuit potential reached a relatively stable state. (3) The impedance spectroscopy (EIS) testing can be conducted, When the potential fluctuation of the open circuit potential is within 10 mV over 2 min. The applied voltage was set to the open-circuit voltage, with an amplitude of 10 mV and a frequency range of 10^−2^ HZ to 10^5^ Hz. (4) After the impedance test, the samples were subjected to polarization testing using linear sweep voltammetry (LSV), with a scanning voltage range of −0.8 V to 1.2 V and a scanning rate of 1 mV/s. Each alloy combination was tested three times.

## 3. Results and Discussion

### 3.1. Microstructural Analysis of CoCrFeMnNiSix HEAs After 900 °C Heat Treatment

**Figure 2:**
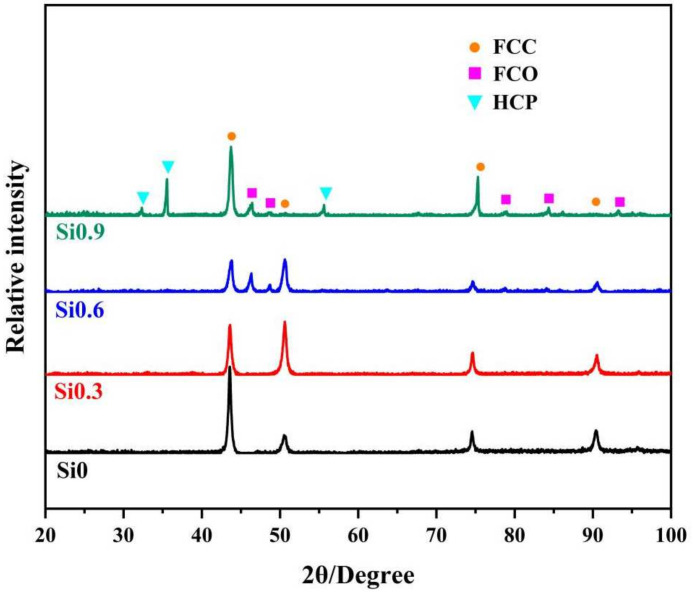
XRD pattern of CoCrFeMnNSix HEAs.

Figure 2 shows the XRD patterns of the CoCrFeMnNiSix HEAs after 900 °C heat treatment. It can be seen that due to the variation in Si content, the position and intensity of the diffraction peaks also change, and the phase composition in the microstructure of the HEAs differs accordingly. When x increases from 0 to 0.3, the phase composition remains a single FCC structure, which is a solid solution in which all elements are uniformly mixed [30]. When x = 0.6, the alloy matrix is still predominantly FCC, but an FCO phase precipitates [31,32]. By comparing the FCO phase here is likely to be Ni-Si. The intensity peaks were at (121), (002), and (430) respectively. When x = 0.9, a new HCP phase appears [33,34,35]. Through comparison and EDS analysis, it was found that the HCP phase is rich in Fe elements. The intensity peaks were at (101), (002), and (003) respectively. After heat treatment, in the HEA with a silicon content of x = 0.9, the intensity of the FCO phase diffraction peak is weaker compared to the HEA with x = 0.6, indicating a reduction in the FCO phase content. This suggests that a relatively high content of Si induces the formation of the FCO phase in the single FCC phase of the CoCrFeMnNi HEA [31]. After quenching at 900 °C, the FCO phase forms in the CoCrFeMnNiSi0.6 HEA, and a new HCP phase appears in the CoCrFeMnNiSi0.9 HEA.

**Figure 3:**
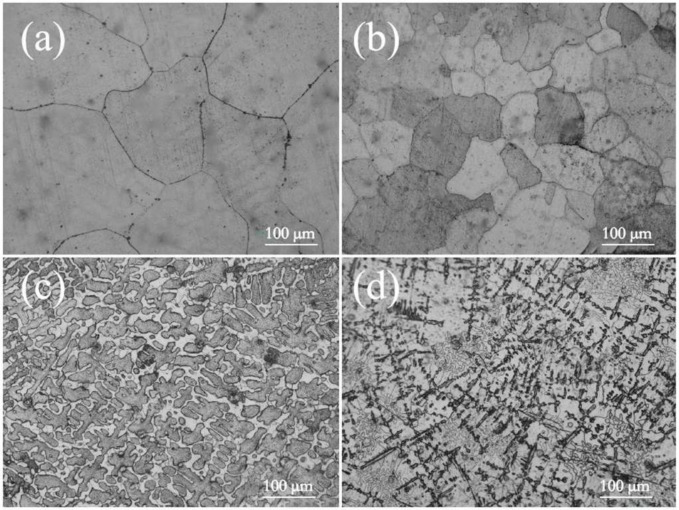
The metallographic microstructure of the CoCrFeMnNSix HEAs: (**a**) x = 0; (**b**) x = 0.3; (**c**) x = 0.6; (**d**) x = 0.9.

Figure 3 shows the optical microstructures of the CoCrFeMnNiSix HEAs after 900 °C heat treatment. In Figure 3a, it can be observed that in the HEA without Si addition, the microstructure transforms from columnar grains to equiaxed grains after the 900 °C heat treatment, and the overall grain size remains relatively coarse. When the Si content is 0.3, the HEA grains are significantly refined (Figure 3b). When the silicon content is 0.6, the HEA microstructure transforms from equiaxed grains to dendritic crystals, with the dendrites connecting to form a network structure that divides the matrix phase (Figure 3c). When the silicon content is further increased to 0.9, the dendrites in the heat-treated HEA become finer compared to when x = 0.6, and the proportion of dendrites in the HEA structure decreases. Some of the dendrites are replaced by black precipitates of varying sizes arranged in a straight heat treatment crisscross pattern, further disrupting the continuity of the HEA matrix (Figure 3d).

**Figure 4:**
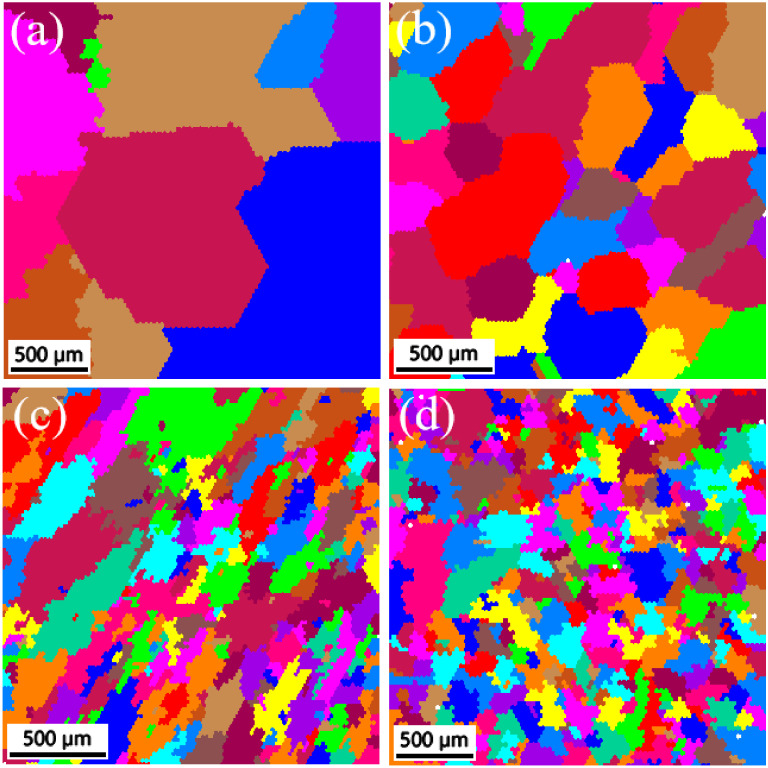
The EBSD grain distribution maps of the CoCrFeMnNiSix HEAs: (**a**) x = 0; (**b**) x = 0.3; (**c**) x = 0.6; (**d**) x = 0.9.

Figure 4 shows the EBSD grain distribution maps of the CoCrFeMnNiSix HEAs after 900 °C heat treatment. When x = 0, the microstructure of the HEA is relatively uniform, regularly shaped, coarse equiaxed grains after heat treatment (Figure 4a). This occurs because, at high temperatures, the increased atomic diffusion at grain boundaries leads to grain coalescence and growth, reducing the number and length of grain boundaries. Subsequently, new grains form and grow at defects such as grain boundaries and dislocations, connecting and rearranging into equiaxed grains [36]. These equiaxed grains have similar dimensions in all directions, which generally results in better mechanical properties compared to columnar grains with a long-axis orientation. When the Si content reaches 0.3, the HEA remains predominantly equiaxed, but the grain size decreases significantly, as demonstrated in Table 1. This is because the added Si disperses in the solid solution and accumulates near the grain boundaries, generating more nucleation sites at high temperatures, leading to the formation of more new grains. After quenching, these refined grains are preserved. As the Si content increases to 0.6, further grain refinement occurs, as seen in Figure 4c, with more fine grains appearing within the larger grains and near the grain boundaries. When the Si content increases further to 0.9, the grain refinement decreased, but more fine grains appeared inside and near the coarse grain boundary, as demonstrated in Figure 4d and Table 1.

**Figure 5:**
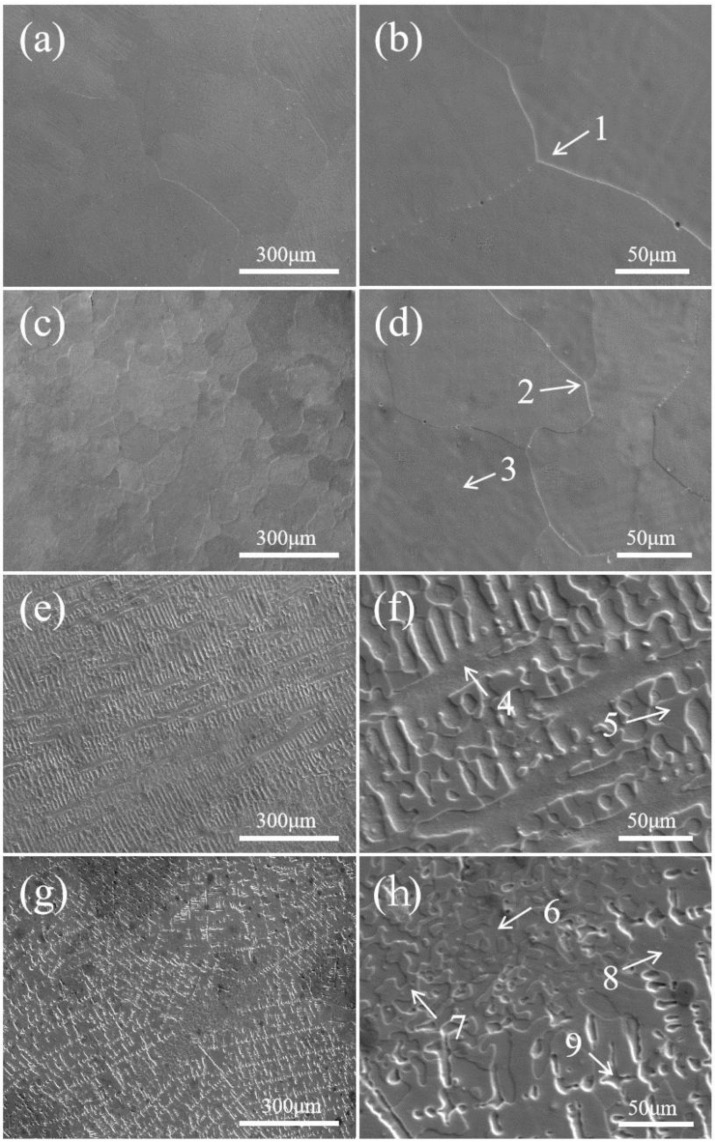
The SEM images of the CoCrFeMnNiSix HEAs: (**a**,**b**) x = 0; (**c**,**d**) x = 0.3; (**e**,**f**) x = 0.6; (**g**,**h**) x = 0.9.

Figure 5 shows the SEM images of the CoCrFeMnNiSix HEAs after 900 °C heat treatment, with images (a–g) magnified 200 times and images (b–h) magnified 1000 times. It can be observed that the equiatomic CoCrFeMnNi HEA without Si addition has uniform grain size. During the annealing process, recrystallization occurs at high temperatures in the columnar grains, leading to changes in the structure and morphology of the metal grains. The overall structure appears as more regular equiaxed grains, as demonstrated in Figure 5a,b.

When the Si content is 0.3, significant grain refinement occurs, mainly due to the rapid cooling of the liquid HEA during the 900 °C water quenching process. As the solid–liquid phase transition occurs, the added Si atoms diffuse into the solid phase, forming smaller solid solution regions. The rapid cooling also increases the nucleation rate of crystals, resulting in reduced grain size, as demonstrated in Figure 5c,d. When the Si content increases to 0.6, the HEA structure completely transforms from equiaxed grains to a dendritic structure, exhibiting a continuous network pattern. This indicates that quenching induces the formation of the dendritic structure.

During the HEA quenching and cooling process, the fine dispersed solid solution particles within the grains hinder grain boundary migration, increasing the curvature of the grain boundaries and promoting the growth and refinement of dendrites, as demonstrated in Figure 5e,f. When the Si content increases to 0.9, the dendrites are slightly refined, and it is believed that a new HCP phase precipitate forms in the alloy microstructure, indicated by the arrow labeled 9 in Figure 5h. This is consistent with results from previous XRD analysis.

**Figure 6:**
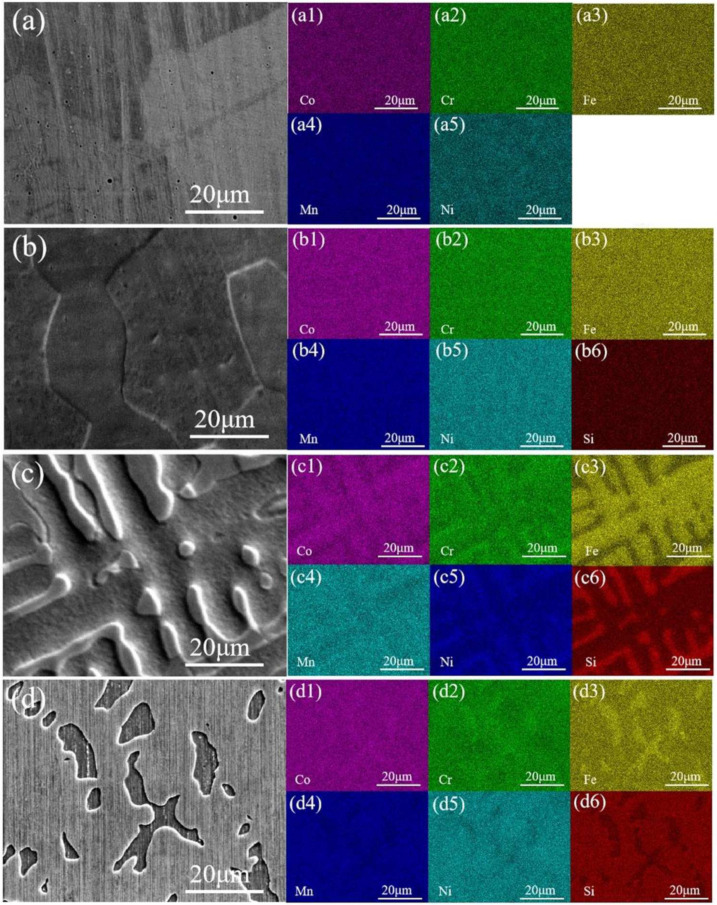
The EDS elemental mapping distribution of the CoCrFeMnNiSix HEAs: (**a**) x = 0; (**b**) x = 0.3; (**c**) x = 0.6; (**d**) x = 0.9.

Figure 6 shows the EDS analysis of the CoCrFeMnNiSix HEAs after heat treatment. As seen in Figure 6(a1–a5), the equiatomic CoCrFeMnNi HEA after 900 °C heat treatment exhibits uniform distribution of Co, Cr, Fe, Mn, and Ni elements throughout the HEA. The actual atomic ratio within the grain in region 1 of Figure 5b is very close to the theoretical value. Combined with the XRD analysis results, the HEA is at the FCC phase. When the Si content is 0.3, the EDS element distribution maps of the CoCrFeMnNiSi0.3 HEA shown in Figure 6(b1–b6), it can be observed that no element aggregation occurs, and the element distribution becomes more uniform. This is because, at sustained high temperatures, the different atomic radii of the principal elements in the HEA hinder cooperative diffusion. Simultaneously, the high temperature increases the diffusion coefficient and raises the strain energy, promoting the thorough diffusion of solute atoms throughout the entire alloy. Elements that had accumulated at the grain boundaries are re-dissolved into the grain interiors [37]. Combined with the XRD analysis results, The HEA phase is still the FCC matrix phase. When the Si content is further increased to 0.6, discontinuous network precipitates appear in the CoCrFeMnNiSi0.6 HEA. As seen in Figure 6(c1–c6), there is enrichment of Co, Cr, and Fe elements within the dendrites, with the specific element proportions shown in Table 2, entry 4, where Fe has the highest concentration within the dendrites. Between the dendrites, Mn, Ni, and Si elements are enriched, with the Si content between the dendrites being significantly higher than within the dendrites. According to the XRD results, an FCO phase has formed. When the Si content is 0.9, Figure 6(d1–d6) and Table 2 indicate that the new phase that appears in the CoCrFeMnNiSi0.9 HEA has the highest Fe content, while the Si content is relatively low. In Figure 5, the post-heat-treatment Regions 5 and 7 and Regions 4 and 6 represent the dendrites and interdendritic areas, respectively. Regions 5 and 7 are enriched in Ni and correspond to the FCO phase. Region 9 is enriched in Fe and corresponds to the HCP phase.

### 3.2. CoCrFeMnNiSix HEAs Mechanical Properties After 900 °C Heat Treatment

After heat treatment of the CoCrFeMnNiSix HEAs, the FCO phase and HCP phase were formed. To investigate the impact of phase changes on the mechanical properties of the CoCrFeMnNiSix HEAs, Vickers microhardness testing, room-temperature compression testing, and room-temperature friction and wear testing were conducted on these HEAs.

**Figure 7:**
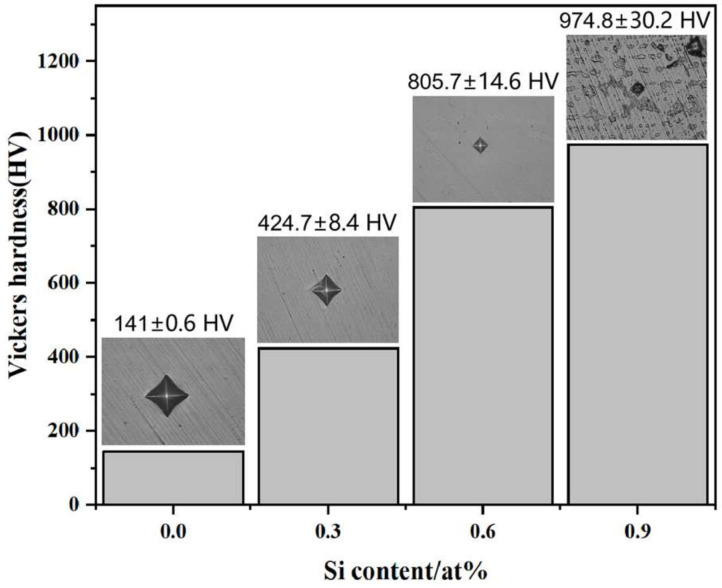
The hardness and indentation morphology of the CoCrFeMnNiSix HEAs.

Figure 7 shows the average Vickers hardness and corresponding indentation morphologies of the CoCrFeMnNiSix HEAs after 900 °C heat treatment. As seen in Figure 7, the Vickers hardness of the HEA generally increases with the addition of Si, and the size of the indentation morphology decreases accordingly. When no Si is added, the hardness is 146.1 ± 0.6 HV, which is slightly lower by 7.8 HV compared to the as-cast HEA [38]. This decrease is attributed to the gradual grain growth during homogenization annealing at 1000 °C, where the equiatomic CoCrFeMnNi HEA exhibits a very uniform solid solution structure, leading to minimal impact from the quenching process. When Si is added to 0.3, significant grain refinement occurs after quenching. During plastic deformation under external forces, the silicon dissolved in the HEA and the grain boundaries impede dislocation slip, increasing the HEA strength. The hardness increases to 424.7 ± 8.4 HV, which is a 190% improvement compared to the HEA without silicon. This increase is due to the accumulation of silicon atoms at the grain boundaries during the 900 °C annealing process, which reduces grain boundary energy and enhances grain boundary migration, leading to grain migration and recrystallization [39]. This is because in HEAs, grain boundaries hinder dislocation movement. During recrystallization, many small grains form in the HEA, and these small grains provide more boundaries, making dislocation movement more difficult, thus increasing hardness [40]. Grain migration during heat treatment eliminates defects and internal stresses within the HEA, further improving its hardness. Additionally, Si slows down the grain’s growth rate, preventing grain coarsening. During the 900 °C quenching process, the rapid cooling effectively suppresses atomic diffusion and grain growth, maintaining grain size within a smaller range. The addition of Si also intensifies the lattice distortion inside the HEA, which increases the HEA hardness by [39]. When Si is added to 0.6, the HEA hardness further increases to 805.7 ± 14.6 HV, representing a 450% improvement over the original HEA. This is not only due to the further refinement of the HEA grains but also due to the appearance of a second phase with a network distribution in the HEA structure segments the HEA, strengthening the HEA’s hardness. When the Si content is increased to 0.9, a new HCP phase appears in the HEA. the HCP phase has more slip systems than FCC phase, but the critical resolved shear stress is higher, thus the slip system is more difficult to be activated, making it less prone to deformation under external forces. Therefore, the HEA has the highest hardness when Si content is increased to 0.9. The dispersed hard FCO phase and Fe rich HCP phase jointly strengthens the HEA, resulting in a continued increase in hardness to 974.8 ± 30.2 HV, which is 667% higher than that of the original HEA.

**Figure 8:**
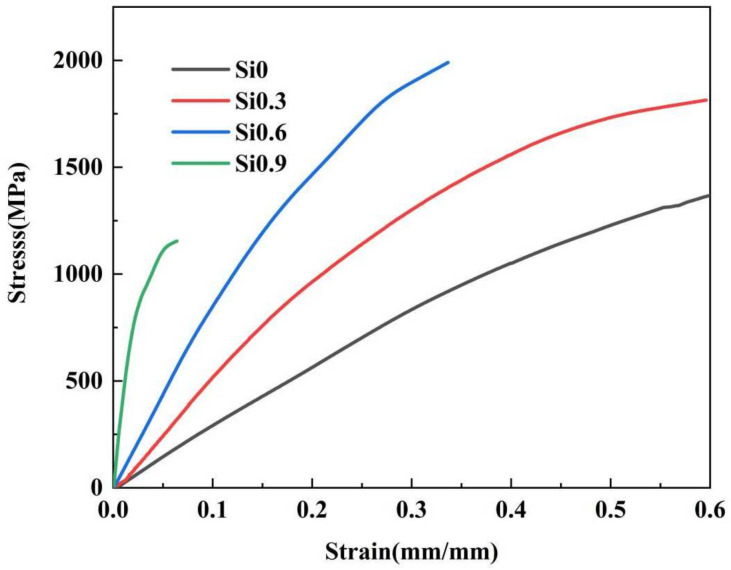
The room temperature compressive stress–strain curves of the CoCrFeMnNiSix HEAs.

Figure 8 shows the stress–strain curves of the CoCrFeMnNiSix HEAs after heat treatment. It can be observed that as the silicon content increases, the slope of the curve also increases. This indicates that the elastic strain modulus of the HEA increases continuously, leading to a continuous improvement in the compressive strength of the HEA for the same amount of deformation. However, the fracture strain of the HEA decreases to varying degrees with the addition of different silicon contents, indicating a reduction in the HEA ductility. According to the compression data in Table 3, the equiatomic CoCrFeMnNi HEA without silicon shows grain growth after heat treatment, the compressive strength is 1367 MPa. When x = 0.3, the addition of Si leads to significant grain refinement, along with the evident effects of solid solution strengthening and grain refinement, resulting in a substantial increase in HEA strength. When the silicon content reaches 0.6, the compressive strength of the HEA continues to rise to 1990.3 MPa, an increase of 45.6% compared to the original HEA. However, the decrease in the fracture strain of the HEA indicates that the HEA’s plasticity is reduced. This is mainly due to the formation of a networked FCO phase in the HEA, which increases dislocation slip resistance during compression, thereby enhancing the strength of the HEA. When x = 0.9, the performance of the HEA deteriorated, both the intensity and the plasticity were substantially decreased. This is because the excessive addition of Si reduces the amount of solid solution in the HEA, thereby diminishing the solid solution strengthening effect. Excessive Si also leads to the formation of brittle HCP phases at grain boundaries, causing grain boundary embrittlement, which in turn reduces the toughness and strength of the HEA. The yield strength of the HEA after heat treatment follows a similar trend as compressive strength, first increasing and then decreasing, reaching a maximum of 1327.5 MPa at x = 0.6, which is 1.7 times that of the silicon free HEA. Overall, the CoCrFeMnNiSix HEAs can achieve multiple strengthening effects, including solid-solution strengthening, grain refinement, and second-phase strengthening after quenching at 900 °C. Adding an appropriate amount of Si to the equiatomic CoCrFeMnNiSix HEA effectively enhances the comprehensive properties of the HEAs. However, for HEAs with higher silicon content, hard and brittle phases are formed after heat treatment, which significantly reduces the toughness of the HEAs.

**Figure 9:**
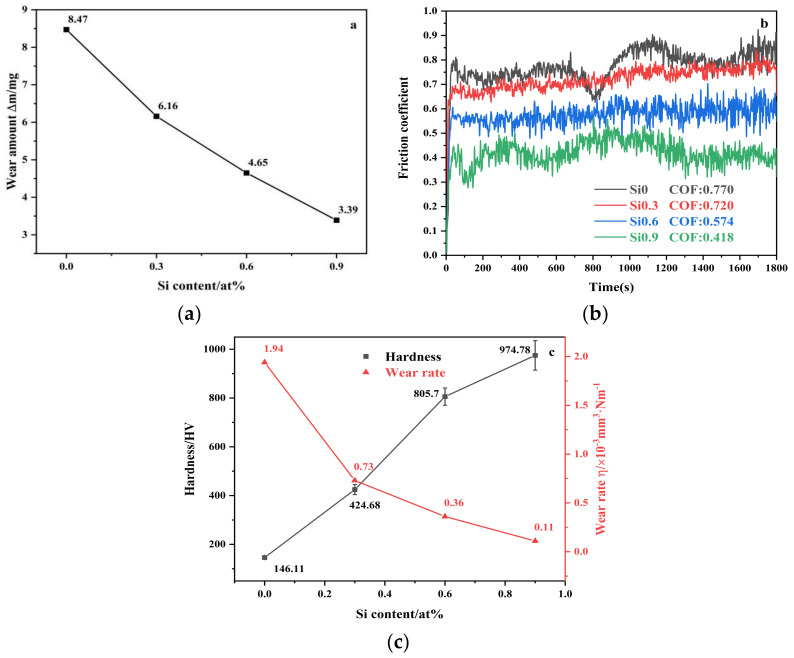
(**a**) Line chart of friction loss mass; (**b**) friction coefficient curve; (**c**) hardness and wear rate relationship line chart of CoCrFeMnNiSix HEAs.

Figure 9 shows the weight loss before and after wear, the friction coefficient versus time curves, and the relationship between hardness and wear rate for CoCrFeMnNiSix HEAs after 900 °C heat treatment. From Figure 9a, it can be observed that the wear loss of the HEA decreases with increasing Si content, showing an overall inverse relationship. To further verify the accuracy of the above conclusion, friction coefficient versus time curves were plotted for these four different Si content HEAs after heat treatment (Figure 9b). The figure shows that the friction coefficient of all HEAs initially increases rapidly, then transitions to a stable or fluctuating state. This is because, at the beginning of the test, the low contact area between the Si3N4 ball and the sliding surface generates high pressure, leading to a rapid increase in the friction coefficient. When the Si content is 0, the friction coefficient of HEA is 0.77. As the Si content increases, the friction coefficient decreases and the wear resistance of the HEA improves, which is consistent with the results obtained from the wear mass loss analysis above. The friction coefficient of the heat treated HEAs is lower compared to that of the as-cast HEAs with the same silicon content [38]. This is attributed to the formation of FCO- and HCP-strengthening phases in Si containing HEAs after high-temperature water quenching. Additionally, the small atomic radius of Si leads to lattice distortion, which results in solid solution strengthening, and the reduced grain size contributes to grain refinement strengthening [41]. Figure 9c illustrates the relationship between HEA hardness and wear rate, showing that as the hardness of the HEA increases significantly from 146.11 HV to 974.78 HV, the wear rate decreases from 2.94 × 10⁻^3^ mm^3^/N·m to 1.1 × 10⁻⁴ mm^3^/N·m. This inverse proportionality between hardness and wear rate is consistent with Archard’s wear theory [42].

After taking the average of multiple measurements, the room temperature friction and wear experiment data for the HEA were obtained. The total distance traveled by the wear ball S was calculated to be 90.5 m. Using the known parameters, the following were calculated [43]:

The formula for calculating the volume change is shown in Equation (1):

(1) $$\Delta V=\frac{\pi Dl^{3}}{6d}$$

where *D* is the wear diameter (mm); *l* is the wear width (mm); and *d* is the wear diameter (mm).

The calculation formula for the wear rate is shown in the following equation:

(2) $$\eta =\frac{\Delta V}{FS}$$

where η is the wear rate; ΔV is the volume change; *F* is the load (N); and *S* is the total friction distance (m).

The calculation formula of volume wear rate and wear resistance is shown in Equation (3):

(3) $$W_{v}=\frac{W_{W}}{\rho }\;\;\;\varepsilon =\frac{1}{W_{v}}$$

where *W_v_* is the volume wear (mm^3^), *W_W_* is the mass wear (g), and *ρ* is the alloy density (g/mm^3^). *ε* is the wear resistance (mm⁻^3^), typically calculated as the inverse of the wear rate, indicating the material’s wear resistance.

As demonstrated in Figure 9c and the wear resistance data in Table 4, the wear rate continuously decreases, and the wear resistance steadily improves with the increase in silicon content in the HEA. This is consistent with the previous results obtained for wear volume and friction coefficient, indicating that the wear resistance of the HEA enhances as the silicon content increases.

**Figure 10:**
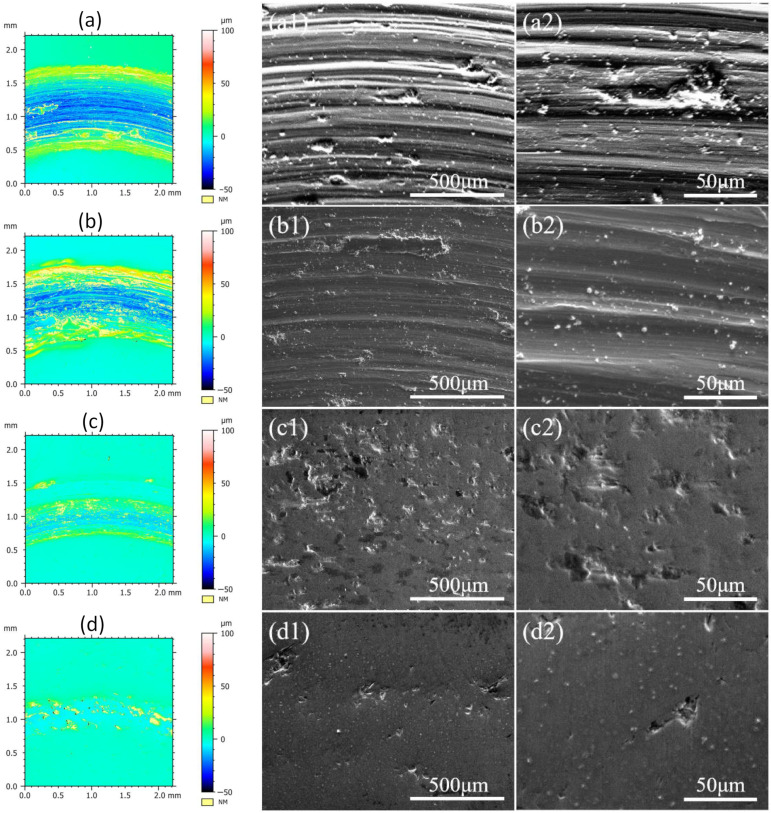
The room temperature friction and wear images of the CoCrFeMnNiSix HEA: (**a**) x = 0; (**b**) x = 0.3; (**c**) x = 0.6; (**d**) x = 0.9.

Figure 10 presents the confocal surface wear track morphology images (a–d) and the SEM images at 100 images (a1–d1) and 500 images (a2–d2) magnification for the CoCrFeMnNiSix HEAs after 900 °C heat treatment. The confocal surface wear track images show that as the Si content increases, the width and depth of the wear tracks continuously decrease, with the deep blue regions representing deep wear tracks gradually shrinking and eventually disappearing. The SEM wear track surface morphology images indicate that after quenching at 900 °C, the HEA without added Si exhibits severe wear, with numerous pits of varying sizes on the surface, the poorest smoothness among all HEAs, and the presence of deep grooves and plowing marks, as seen in Figure 10(a1,a2). As the Si content increases, the surface becomes increasingly smooth and the wear resistance improves. When x = 0.9, the surface is the smoothest, with only a few plowing marks and wear debris, the fine grooves nearly disappear and only slight abrasive wear occurs, resulting in the best wear resistance, as demonstrated in Figure 10(d1,d2). These results are consistent with the previous friction data, demonstrating that heat treatment effectively enhances the friction and wear performance of silicon containing HEAs. Among this series of HEAs, the CoCrFeMnNiSi0.9 HEA exhibits the best wear resistance after 900 °C heat treatment.

### 3.3. Corrosion Resistance of CoCrFeMnNiSix After 900 °C Heat Treatment

**Figure 11:**
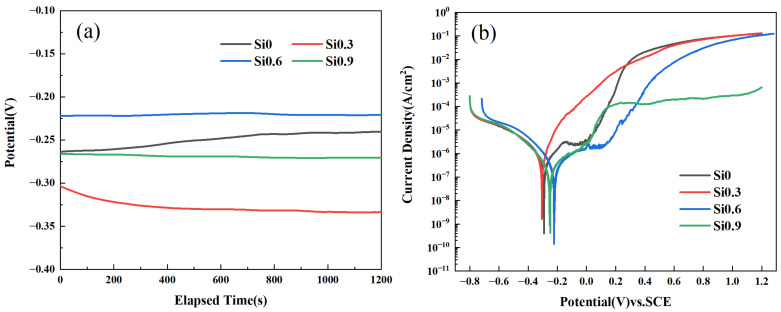
The CoCrFeMnNiSix HEAs in 3.5 wt.% NaCl corrosion solution: (**a**) open circuit potential (OCP); (**b**) potentiodynamic polarization curves.

Figure 11 shows the open circuit potential and potentiodynamic polarization curves of the CoCrFeMnNiSix HEA samples after 900 °C heat treatment in a 3.5 wt.% NaCl solution. Figure 11a indicates that the open circuit potential of the HEA samples stabilizes after being left in the corrosive solution for 30 min before impedance and potentiodynamic polarization testing. Figure 11b presents the potentiodynamic polarization curves of this series of HEAs after 900 °C heat treatment, where the polarization curves for x = 0, x = 0.6, and x = 0.9 HEAs show a distinct passivation region, while the x = 0.3 HEA does not exhibit a clear passivation region.

To quantitatively compare the corrosion resistance of each HEA, the corrosion rates for each HEA was calculated using Equations (4) and (5) and are listed in Table 5.

(4) $$K_{corr}=\frac{i_{corr}\cdot k\cdot EW}{\rho }$$

Where *i_corr_* is the corrosion current density (A·cm⁻^2^), *ρ* is the mass density (g·cm⁻^3^), *EW* is the equivalent weight of the electrode (g), *k* is a constant with a value of 3272 mm/(A·cm·year), and *Kcorr* is the corrosion rate, expressed in millimeters per year (mmpy). The *EW* is calculated as the weighted average of the ratio of the atomic weight of each element in the HEA to the number of electrons exchanged during the electrochemical reaction.

(5) $$EW=\sum \frac{f_{i}n_{i}}{A_{i}}$$

where *f_i_* is the mass fraction of the *i*-th alloy component, *n_i_* is the number of exchanged electrons for the *i*th alloy component, *A_i_* is the atomic weight of the *i*th alloy component (g/mol).

The pitting potential for the x = 0.6 HEA is 157.649 mV as demonstrated in Table 5, higher than other HEAs. This indicates that the HEA with x = 0.6 has the best stability of its passivation film. The table also shows that when the Si content is 0.6, the HEA has the highest self corrosion potential and the lowest self corrosion current, suggesting that this HEA has the best corrosion resistance. This is because the FCO phase is rich in Ni acts as the anode and is preferentially corroded, while the FCC matrix phase serves as the cathode and is protected. The FCO phase dissolves preferentially compared to the FCC phase in 3.5 wt.% NaCl solution [44]. When x = 0.3, the HEA has the lowest self corrosion potential and the highest self corrosion current, indicating the poorest corrosion resistance. This may be due to grain refinement in the HEA, leading to an increase in grain boundaries, where the higher strain energy at the grain boundaries easily induces pitting corrosion and breakdown on the HEA surface [45,46]. Based on the corrosion rate (*Kcorr*) of the HEAs listed in Table 4, the corrosion resistance of the HEAs can be ranked in descending order as x = 0.6 > x = 0.9 > x = 0 > x = 0.3.

**Figure 12:**
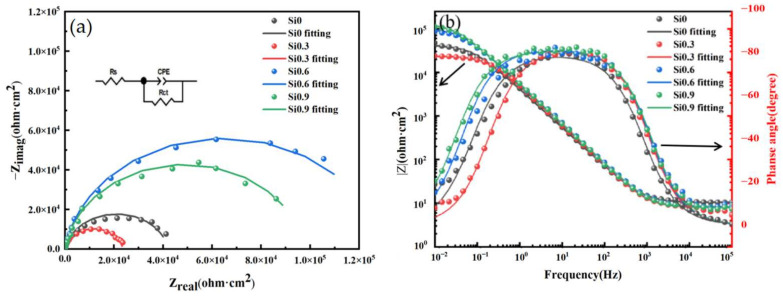
The EIS images of the CoCrFeMnNiSix HEA: (**a**) Nyquist plot and equivalent circuit diagram; (**b**) Bode plot.

Figure 12 shows the EIS of CoCrFeMnNiSix HEAs after 900 °C heat treatment in a 3.5 wt.% NaCl corrosion solution. The equivalent circuit is shown in Figure 12a. The solid line represents the curve simulated using Zview (3.1) software, while the points represent the original data. It can be observed that the simulated curve closely matches the original data. Based on the analysis of potentiodynamic polarization curves and impedance spectra, the equivalent circuit diagram was fitted (Figure 12a). In this diagram: RS represents the resistance of the solution, Rct represents the charge transfer resistance, which is the impedance controlled by the kinetics of the electrochemical reaction, The constant phase element (CPE) represents the double layer capacitance at the electrolyte solution electrode interface and is used to compensate for the effects caused by the non uniform distribution of current and potential in the system [47]. The impedance of the CPE, $Z_{CPE}$ is defined by the following equation [48]:

(6) $$Z_{CPE}=\frac{1}{{\left(J\omega \right)}^{n}Y_{o}}$$

where *Y_O_* is the proportional factor, typically ranging between 0.5 and 1, reflecting the deviation of the double layer capacitance from an ideal capacitor. The closer the value of *Y_O_* is to 1, the more similar the behavior of the CPE is to that of an ideal capacitor, indicating that the system is closer to behaving as an ideal capacitor.

In the Nyquist plot of Figure 12a, it can be observed that the system exhibits an incomplete semicircular capacitive loop in the high frequency region. This indicates that a large number of charge particles are continuously transferred at the uneven electrode interface in contact with the corrosive solution. The radius R of the circle where this capacitive loop is located is closely related to the state of the corroded surface and the composition of the substances responsible for charge transfer. The larger the R value, the higher the impedance of the material, and the better its corrosion resistance. Therefore, using the R value as the criterion for evaluating corrosion resistance, the CoCrFeMnNiSix HEAs can be ranked in descending order as x = 0.6 > x = 0.9 > x = 0 > x = 0.3, which is consistent with the results obtained from the potentiodynamic polarization curves discussed earlier. From the relationship between impedance |Z| and frequency in Figure 12b, it can be seen that in the high frequency region at 105 Hz, the solution resistance Rs of the HEAs shows little difference. In the low frequency region at 10^−2^ Hz, the polarization resistance Rct of the CoCrFeMnNiSi0.6 HEA is the highest, reaching 12,915 Ω. This indicates that this HEA has superior corrosion resistance. This result is consistent with the Nyquist plot shown in Figure 12a and the potentiodynamic polarization curves in Figure 12b. Except for the x = 0.3 HEA, the slopes of the other curves are close to −1, indicating that a passivation film has formed on the surface of these HEAs. From the relationship between phase angle and frequency in Figure 12b, it is evident that the x = 0.3 HEA has a lower phase angle in the low frequency region and a narrower phase angle plateau in the mid frequency region, indicating that passivation film of the HEA is the least stable. In contrast, the other HEAs have higher phase angles in the low frequency region and better phase angle broadening in the mid frequency region, suggesting that their passivation films are relatively stable. Combined with the data in Table 6, it can be seen that the Y0 value in the CPE for the x = 0.3 HEA is higher than that of the x = 0 HEA, indicating a decrease in the passivation density of the film for the x = 0.3 HEA. The Y0 value decreases as the silicon content increases, indicating that the passivation density of the film improves with increasing Si content, which is consistent with the previous conclusions. The n values in the CPE are all slightly less than 1, indicating that the double layer capacitance in this experiment is close to an ideal capacitor. Moreover, the n value increases with the Si content, suggesting that the increased Si content in the HEA enhances the protective effect of the passivation film [49].

**Figure 13:**
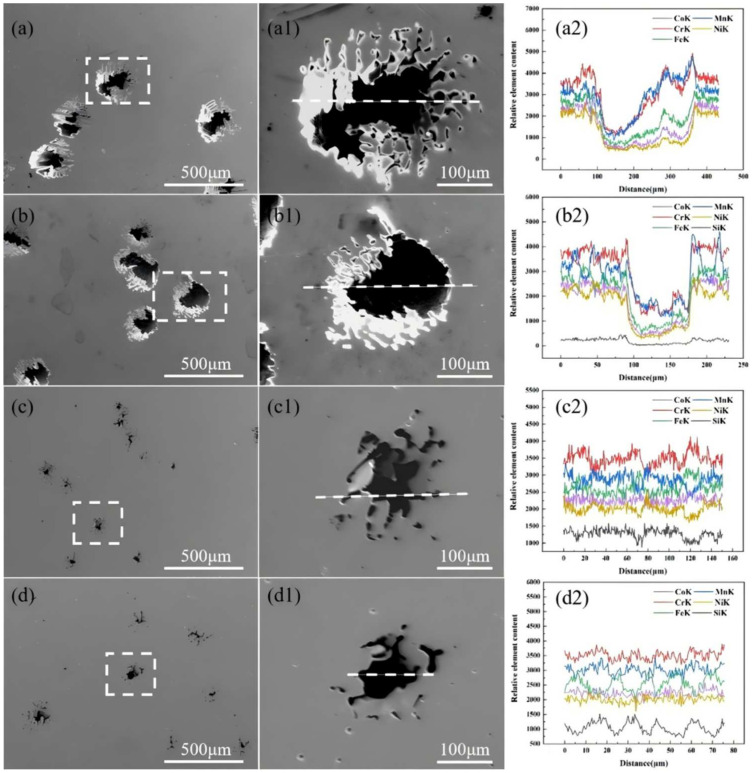
The surface pit morphology and EDS line scan images of the CoCrFeMnNiSix HEA after corrosion in 3.5 wt.% NaCl solution: (**a**) x = 0; (**b**) x = 0.3; (**c**) x = 0.6; (**d**) x = 0.9.

Figure 13 shows the SEM images of CoCrFeMnNiSix HEAs after 900 °C heat treatment and subsequent corrosion, along with the EDS line scan element content curves at different points on the surface pits. High temperature heat treatment effectively eliminates elemental segregation and macro residual stress within the HEA. The SEM images reveal that the electrochemically corroded surfaces of the HEAs exhibit pitting of varying sizes and quantities, with the pits being irregularly elliptical and the surrounding areas relatively smooth, as demonstrated in Figure 13. The surface of the silicon-free HEA exhibits larger and deeper pits, with a network-like pattern around the pits. When the silicon content is 0.3, deeper corrosion pits appear, surrounded by numerous bright white smaller pits, indicating a reduction corrosion resistance of the HEA. The corrosion surfaces of the x = 0.6 and x = 0.9 HEAs show a transition from relatively fewer, larger, and deeper pits to relatively more, smaller, and shallower pits, suggesting that the corrosion resistance of these two HEAs has improved. From the magnified corrosion surface morphology images (a1–d1) and the corresponding EDS line scan element content curves (a2–d2) of the HEAs, it can be seen that in the x = 0 HEA, the content of Cr and Mn elements is higher in the strongly corroded areas (Figure 13(a2)). This is because the addition of Mn during corrosion consumes Cr based oxides in the passivation film [50], leading to instability in the passivation structure and an increase in current density. When the Si content in the HEA is 0.3, the overall content of all elements decreases without selectivity, as seen in Figure 13(b2). This due to two factors: first, the presence of Mn depletes the Cr oxide passivation film that protects the HEA; second, the addition of Si refines the HEA grains, creating more grain boundaries high energy line defects that can easily induce pitting. In the x = 0.6 HEA, Si elements are enriched in the strongly corroded areas (Figure 13(c2)), possibly because Si and Mn form stable amorphous MnO-SiO_2_ during corrosion, which reduces the impact of Mn on the Cr based passivation film and hinders Cl⁻ ions from attacking the matrix, thereby enhancing the corrosion resistance of the HEA. In the elemental distribution map of the x = 0.9 HEA, as seen in Figure 13(d2), Fe, Si, and Mn elements alternately increase and decrease, suggesting that the Fe-rich HCP phase and the Si- and Mn-rich FCO phase act as micro-cathodes, protecting the FCC matrix phase, which serves as the anode.

## 4. Conclusions

(1) After the 900 °C heat treatment, the crystal structure and microstructure of the CoCrFeMnNiSix HEAs underwent significant changes. When the silicon content x is 0 and 0.3, no new phase is formed and the matrix remains a FCC phase, but the grain structure transitions from irregular columnar grains to more regular equiaxed grains. When x = 0.6, a FCO phase rich in Ni, and Si is formed. Where the microstructure transforms from equiaxed grains to a continuous network dendritic structure. When x = 0.9, the heat-treated HEA shows the presence of both an FCO phase and a HCP phase rich in Fe, with the dendrites in the microstructure becoming finer and less abundant and the HCP phase appearing as a discontinuous network structure.

(2) After 900 °C heat treatment, the hardness of the CoCrFeMnNiSix HEAs increases steadily with the addition of Si. When the silicon content reaches 0.9, the hardness of the HEA peaks at 974.8 ± 30.2 HV, which is approximately 6.7 times higher than that of the silicon-free HEA. For Si contents ranging from 0 to 0.6, the compressive strength and yield strength of the HEA increase steadily due to the combined effects of solid solution strengthening, grain refinement strengthening, and second-phase strengthening, while its ductility slightly decreases. However, when the silicon content reaches 0.9, the increase in brittle phases within the HEA leads to a significant decrease in both strength and ductility. The CoCrFeMnNiSi0.6 HEA exhibits the highest compressive strength and yield strength, reaching 1990.3 MPa and 1327.5 MPa, respectively, representing increases of approximately 65% and 46% compared to the HEA without Si. The wear resistance of the HEAs improves with increasing Si content, with a positive correlation between hardness and wear resistance, consistent with Archard’s theory. Among these HEAs, the HEA with x = 0.9 has the smoothest surface after friction testing and the best wear resistance, reaching 0.21 mm⁻^1^.

(3) Among the CoCrFeMnNiSix HEAs after 900 °C heat treatment, the HEA with x = 0.6 has the lowest self corrosion current density at 0.23 μA/cm^2^ and the highest pitting potential at 157.65 mV, indicating the best corrosion resistance. This is mainly because the newly formed FCO precipitate phase acts as a sacrificial anode, being preferentially corroded and thereby reducing the likelihood of corrosion in the FCC matrix phase. The corrosion resistance of the HEAs after heat treatment ranks in descending order as x = 0.6 >x = 0.9 > x0 > x = 0.3.

## Figures and Tables

**Table 1:** Average grain size of CoCrFeMnNiSix HEAs after 900 °C heat treatment.

Silicon Content	0	0.3	0.6	0.9
Average grain size (μm)	1141.94	393.25	249.96	203.25

**Table 2:** Elemental distribution (at.%) in microregions of CoCrFeMnNiSix HEAs.

Si Contents	Regions	Elements					
		Co	Cr	Fe	Mn	Ni	Si
0	1	19.89	19.68	20.74	19.34	20.35	/
0.3	2 3	19.07 19.25	19.04 19.48	19.66 19.22	19.05 19.44	19.45 19.27	3.74 3.35
0.6	4 5	19.45 14.08	19.15 15.58	20.60 14.87	16.34 18.96	17.68 21.25	6.78 15.26
0.9	6 7 8 9	19.47 15.22 13.66 14.74	18.03 16.23 13.85 18.74	21.17 15.59 10.98 28.87	16.86 17.64 20.81 14.83	16.86 19.55 20.10 14.19	7.62 15.76 20.60 9.62

**Table 3:** Compression properties of CoCrFeMnNiSix high entropy.

Silicon Content	0	0.3	0.6	0.9
Breaking strain	>60%	>60%	33.7%	6.4%
Yield strength/MPa	805.3	1246.9	1327.5	841.5
Compressive strength/MPa	1367	1813.8	1990.3	1154.3

**Table 4:** Parameters for the room temperature friction and wear test of the CoCrFeMnNiSix HEAs.

Si Content	0	0.3	0.6	0.9
Wear mark width l/mm	1.96	1.42	1.13	0.75
Wear mark diameter D/mm	8.01	7.92	7.85	7.78
Volume wear/mm^3^	5.26	1.98	0.99	0.29
Wear resistance/mm^−1^	0.11	0.13	0.16	0.21

**Table 5:** Electrochemical corrosion parameters of CoCrFeMnNiSix HEAs.

Si Content	Ecorr (mV)	Icorr (μA/cm^−2^)	Epit (mV)	Kcorr (mm/year)
0	−262.620	0.765	21.408	0.01996
0.3	−333.943	1.276	10.188	0.03171
0.6	−213.627	0.230	157.649	0.00320
0.9	−289.757	0.342	44.250	0.00961

**Table 6:** The equivalent circuit parameters of the EIS for the CoCrFeMnNiSix HEAs.

Si Content	Rs (ohm)	Rct (ohm)	CPE	
			Y0 (F/cm^2^)	N
0	9.460	42,145	4.6156 × 10^−5^	0.89
0.3	8.083	23,563	5.6727 × 10^−5^	0.91
0.6	8.239	1.2915 × 10^5^	4.4024 × 10^−5^	0.94
0.9	7.986	97,547	3.5206 × 10^−5^	0.93

## Data Availability

Data are contained within the article.
